# A higher ratio of IL-2/IL-4 may be an early predictor of acute graft-versus-host disease after allogeneic hematopoietic stem cell transplantation

**DOI:** 10.3389/fimmu.2025.1620761

**Published:** 2025-07-11

**Authors:** Yu Fu, Meifang Zhao, Xiaoliang Liu, Sujun Gao, Yehui Tan

**Affiliations:** Department of Hematology, The First Hospital of Jilin University, Changchun, China

**Keywords:** allogeneic hematopoietic stem cell transplantation, acute graft-versus-host disease, cytokines, diagnosis, therapeutic efficacy

## Abstract

**Objectives:**

To investigate the correlation between cytokines at different time points after allogeneic hematopoietic stem cell transplantation (allo-HSCT) and the onset, severity, and therapeutic efficacy of acute graft-versus-host disease (aGVHD).

**Methods:**

We performed a retrospective analysis of patients who underwent allo-HSCT from January 2019 to December 2021. Patients were divided into a training (first two years) and validation cohort (third year). Serum cytokines levels (TNF-α, IL-2, IL-4, IL-6, IL-10) on days +7, +14, +21, and +28 were measured and compared between patients developed aGVHD and those did not. Clinical characteristics were analyzed. Training cohort results were verified in the validation cohort to identify potential predictive markers for aGVHD.

**Results:**

The training cohort included 89 patients who underwent allo-HSCT, in which 29 patients developed aGVHD. Forty patients were enrolled in the validation cohort and 17 patients suffered aGVHD. Significant differences were observed in the doses of infused CD34^+^ and mononuclear cells between the two cohorts, whereas other baseline clinical characteristics were comparable. In the training set, the ratio of IL-2/IL-4 ≥1.103 on day +7 associated with an 8.87-fold increased risk of aGVHD. After excluding sepsis and engraftment syndrome cases, the IL-2/IL-4 ratio on day +7 remained associated with aGVHD. Under these conditions, IL-2/IL-4 ≥0.989 on day +7 suggested a 5.875-fold increased aGVHD risk. The validation set confirmed IL-2/IL-4 as an early and reliable aGVHD indicator. Among the 29 patients with aGVHD in the training set, 17 had grade I and 12 had grade II–IV aGVHD. TNF-α (day +7) and IL-2 (day +28) significantly increased in grade I aGVHD. After excluding sepsis and ES cases, 19 had aGVHD (12 grade I and 7 grade II–IV aGVHD). No cytokine was significantly associated with aGVHD severity. Twenty-two of 29 patients received corticosteroids as first-line treatment; the complete remission (CR) rate was 68.2% (15/22). Subgroup analysis revealed cytokines were comparable between patients achieved CR and those did not.

**Conclusions:**

A higher IL-2/IL-4 ratio on day +7 may be an early predictive biomarker of aGVHD onset. Nevertheless, whether these five cytokines could predict aGVHD severity or therapeutic efficacy remain unclear.

## 1 Introduction

Allogeneic hematopoietic stem cell transplantation (allo-HSCT) is potentially curative for hematopoietic malignancies. Acute graft-versus-host disease (aGVHD), alongside infection and relapse, substantially affect allo-HSCT success (1). aGVHD follows a three-step immune process, with its pathogenesis recently reviewed in detail (2, 3). It begins with host antigen-presenting cells (APCs) activation owing to conditioning-induced host tissue injury. Donor T cells are activated, triggering inflammatory cytokines release, with subsequent proliferation of alloreactive T cells, resulting in host damage and further inflammation (2, 4, 5). This is followed by activation and proliferation of effector T-lymphocytes which eventually lead to recruitment and activation of additional mononuclear effectors and amplification of the “cytokine storm” (6).

Classic aGVHD occurs within 100 d post-transplant, typically affecting skin, gut, and liver (7, 8). Despite the standard prophylactic strategy, 40%–60% of allo-HSCT recipients develop aGVHD (9). Clinically, aGVHD severity is scored using a composite grade (I–IV) based on skin, upper and lower GI tract, and liver clinical symptom stages. Treatment is initiated according to the grades. Grade I aGVHD is managed with close observation and topical treatment, whereas grade II–IV aGVHD requires immediate systemic corticosteroids treatment as the standard first-line therapy, with a response rate of 40%–50%, and approximately 50% of patients develop steroid-refractory (SR) aGVHD. High-dose systemic glucocorticoids may cause multiple acute and chronic toxicities, including infection, immune suppression, disease relapse, and metabolic complications (10, 11). SR aGVHD portends a poor prognosis, with <30% long-term survival (3, 12–17), and early intervention may improve survival outcomes.

Normally, aGVHD diagnosis depends on clinical symptoms and pathological biopsy, which lacks specificity and clinical feasibility. A higher CD4/CD8 ratio in bone marrow allogeneic grafts increases grade II–IV aGVHD risk (18); however, this may not apply to recipients who received only peripheral blood grafts. A predictive model established by 27 indices (8 cytokines, 19 conventional biochemical markers) may predict aGVHD onset earlier (19); nevertheless, excessive indices are complex. Besides, several potential serum biomarkers are identified, including IL-6, ST2, IL-2Rα, HGF, TNFR1, IL-8, and TIM3 (20, 21). These biomarkers predict the response rate and prognosis better than the standard Grade I-IV clinical severity scale (22); however, it is costly. Therefore, exploring precise, noninvasive, and convenient monitoring indicators for early aGVHD detection and treatment response prediction is crucial. aGVHD is an alloreactive T cell-mediated systemic inflammatory disorder, with cytokines as potential biomarkers for diagnosis and prognosis is feasible (8, 20, 23); however, which and how cytokines affect aGVHD remains unclear. Therefore, this study explores the correlation of serum cytokines at different time points and aGVHD-related events (onset, grading and steroid sensitivity) after allo-HSCT, aiming to provide insights for early diagnosis and treatment, thereby improving the overall efficacy and survival outcomes of aGVHD.

## 2 Patients and methods

### 2.1 Patients

In total, 129 patients were enrolled and treated at our center from January 2019 to December 2021. Eighty-nine cases from the first 2 years were assigned to the training set, while the remaining patients were assigned to the validation set. Inclusive criteria included (1): patients who received myeloablative conditioning (MAC) regimen and underwent allo-HSCT (2); patients who achieved successful hematopoietic engraftment; and (3) serum cytokines detected regularly after allo-HSCT. Exclusive criteria included (1): patients who died within 30 d from complications such as infection and conditioning regimen toxicity rather than aGVHD (2); patients who failed to achieve hematopoietic engraftment (3); patients who received reduced-intensity conditioning regimens due to advanced age or organ dysfunction (4); patients who received enhanced conditioning regimens and underwent salvage allo-HSCT; or (5) patients who relapsed early and quickly reduced immunosuppressive agents.

Clinical characteristics, such as gender, age, diagnosis, type of transplantation, HCT-CI and EBMT scores, conditioning regimens, CD34^+^cells, mononuclear cells (MNC), stem cell sources, engraftment, aGVHD prophylaxis, and aGVHD-related events (aGVHD onset, severity, and treatment), were collected. The correlation between serum cytokines (TNF-α, IL-2, IL-4, IL-6, IL-10) levels at different points after allo-HSCT (day +7, +14, +21, and +28) and aGVHD-related events were explored. Subgroup analysis were conducted to analyze the effect of sepsis and engraftment syndrome (ES) on cytokine levels in patients with aGVHD.

This study was approved by the ethics committee of The First Hospital of Jilin University (approval number:2024-674), and conducted in accordance with the Declaration of Helsinki. All participants provided written informed consent before enrollment. All data were de-identified, and no person information appeared in this paper.

### 2.2 Conditioning regimens and GVHD prophylaxis

All the patients received MAC regimens. For patients diagnosed with leukemia or myelodysplastic syndrome (1): mBuCy for MSD-HSCT: hydroxyurea (Hu; 80 mg/kg/d, divided twice, orally (PO), day -10), cytarabine (Ara-C; 2 g/m^2^/d, intravenously (IV), day -9), busulfan (Bu; 3.2 mg/kg/d, administered in four doses, IV, days -8 to -6), cyclophosphamide (CTX; 1.8 g/m^2^/d, IV, days -5 and -4), and semustine (MeCCNU; 250 mg/m^2^/d, PO, day -3) (2); mBuCy for haplo-HSCT: Ara-C (4 g/m^2^/d, IV, days -10 to -9), with the same Bu, CTX, and MeCCNU dosages as MSD-HSCT (3); mCy/TBI+Ara-C for UCBT or patients with T-ALL and incompletely eradicated tumor masses: total body irradiation (TBI; 600 cGy, divided twice, days -7 and -6), Ara-C (2 g/m^2^, q12h, IV, days -5 and -4), G-CSF (5 µg/kg/d, subcutaneous, days -6 to -4), CTX (60 mg/kg/d, IV, days -3 and -2), and MeCCNU. For patients with aplastic anemia (1): Cy+ATG for MSD-HSCT: CTX (50 mg/kg/d, IV, days -5 to -2), rabbit anti-thymocyte globulin (rATG; 2.5 mg/kg/d, IV, days -5 to -2); and (2) mBuCy+ATG for haplo-HSCT: Bu (3.2 mg/kg/d, administered in four doses, IV, days -7 to -6); CTX and ATG doses were same as MSD-HSCT.

All patients received cyclosporine (CsA; 1.5 mg/kg, q12h, IV, from day -9, then 3–5 mg/kg/d, PO, after bowel function normalized), Mycophenolate mofetil (MMF, 30 mg/kg/d divided twice, PO, from day -9, reduced by half dosage post-engraftment, and discontinued on day +30). Methotrexate (MTX; 15 mg/m^2^/d, IV, on day +1, reduced to 10 mg/m^2^/d on days +3, +6, and/or +11). rATG from days -5 to - 2 was administered intravenously at a total dose of 10 mg/kg for haplo-HSCT or URD-HSCT and 4.5 mg/kg for partial MSD-HSCT.

### 2.3 Grading and evaluation of aGVHD

aGVHD grading was based on the modified Glucksberg and Mount Sinai Acute GVHD International Consortium criteria. Response criteria were defined as described in the EBMT−NIH−Center for International Blood and Marrow Transplant Research (CIBMTR) Task Force position statement.

### 2.4 Cytokines detection

Peripheral blood samples (3 mL) were collected in ethylene diamine tetraacetic acid (EDTA)-containing vacuum tubes at different time points (days +7, +14, +21, and +28). The serum was separated within 24 h. Flow cytometry measured TNF-α, IL-2, IL-4, IL-6, and IL-10 (pg/mL) levels.

### 2.5 Statistical analyses

All statistical analyses were conducted using the Statistical Package for Social Science (SPSS, version 23.0) and GraphPad Prism (version 10.0). Spearman’s correlation for bivariate samples was used for correlation analyses and continuous variables were compared using non-parametric tests (Kruskal–Wallis one-way analysis of variance/Mann–Whitney U-test). χ2 or Fisher’s exact test was used to compare categorized variables. A Receiver Operating Characteristic (ROC) curve determined the cut-off value of cytokines. Logistic regression was used to analyze the correlation of serum cytokine levels with aGVHD-related events (onset, grading, and steroid sensitivity). A P-value <0.05 was considered statistically significant.

## 3 Results

### 3.1 Clinical characteristics

The training set consisted of 89 patients retrospectively analyzed from January 2019 to December 2020, including 49 males and 40 females, with a median age of 34 years (range, 2.7–61). Grafts were derived from haploidentical donors (HID, n = 63), matched sibling donors (MSD, n =18), matched unrelated donors (MUD, n = 6), or unrelated cord blood donors (UCBD, n = 2). The median doses of infused CD34^+^cells and MNC were 3.6 (range, 0.1–8.2) × 10^6^/kg and 8.3 (range, 0.2–18.3) × 10^8^/kg, respectively. Neutrophil and platelet engraftment occurred on days +14 (range, 10–27) and +15 (range, 10–45). Nineteen patients had sepsis and 10 had ES. Twenty-nine of 89 patients developed aGVHD, whereas 60 did not.

A validation set was established with the same inclusive and exclusive criteria as the training set. Forty patients from January 2021 to December 2021 were enrolled, including HID-HSCT, MSD-HSCT, MUD-HSCT, and UCBT were 34, 4, 1 and 1 cases, respectively. The median age was 28.7 (range, 9–57) years, with 52.5% were male. The median doses of infused CD34^+^cells and MNC were 4.3 (range, 0.2–9.0) ×10^6^/kg and 6.2 (range, 0.3–11.4) ×10^8^/kg, respectively. Neutrophil and platelet engraftment occurred on day +14 (range, 11–21) and day +15 (range, 12–40). Six patients had sepsis and 13 cases with ES, and 17 cases with aGVHD in the validation cohort. Significant differences were observed in the infused CD34^+^ cells and MNC doses between the training and validation cohorts (P = 0.009 and P = 0.003, respectively), while other clinical characteristics were similar in these two groups (all P >0.05, **Table 1**).

**Table 1:** Characteristics of patients included in this study.

Characteristics	Training set	Validation set	*P* value
Gender, male (%)	49 (55.0)	21 (52.5)	0.788
Age, median (range), y	34 (2.7–61)	28.7 (9–57)	0.076
Diseases, n (%)			0.742
AML	40 (45.0)	18 (45.0)	
ALL	22 (24.7)	12 (30.0)	
MDS	14 (15.7)	3 (7.5)	
CML	8 (9.0)	1 (2.5)	
AA	5 (5.6)	6 (15.0)	
Donor type, n (%)			0.088
HID	63 (70.8)	34 (85.0)	
MSD	18 (20.2)	4 (10.0)	
MUD	6 (6.8)	1 (2.5)	
UCBD	2 (2.2)	1 (2.5)	
Stem cell source, n (%)			0.878
PB+BM	75 (84.3)	34 (85.0)	
PB	12 (13.5)	5 (12.5)	
CB	2 (2.2)	1 (2.5)	
Neutrophil engraftment, median (range), d	14 (10–27)	14 (11–21)	0.815
Platelet engraftment, median (range), d	15 (10–45)	15 (12–40)	0.758
CD34^+^cells, median (range), ×10^6^/kg	3.6 (0.1–8.2)	4.3 (0.2–9.0)	0.009
MNC, median (range), ×10^8^/kg	8.3 (0.2–18.3)	6.2 (0.3–11.4)	0.003
AGVHD, n (%)			0.279
Yes	29 (32.6)	17 (42.5)	
No	60 (67.4)	23 (57.5)	
AGVHD grade, n (%)			0.422
I	17 (58.6)	12 (70.6)	
II–IV	12 (41.4)	5 (29.4)	

AML, acute myeloid leukaemia; ALL, acute lymphoid leukaemia; MDS, myelodysplastic syndrome; CML, chronic myeloid leukaemia; AA, aplastic anemia; HID,haploidentical donors; MSD, matched sibling donors; MUD, matched unrelated donors; UCBD, unrelated cord blood donors; PB, peripheral blood; BM, bone marrow; CB, cord blood; MNC, mononuclear cells; aGVHD, acute graft-versus-host disease.

### 3.2 Outcomes

#### 3.2.1 Correlation between serum cytokines and aGVHD onset

In the training set, 29 patients developed aGVHD (10 males and 19 females, median age 27 (range, 4–58) years, and median onset time 32 (range, 15–61) days). Among these patients, five patients had sepsis and five had ES simultaneously. The remaining 60 patients without aGVHD comprised 39 males and 21 females. Their median age was 34.5 (range, 2.7–61) years. In the non-aGVHD group, 14 patients had sepsis, and 5 had ES. The median time to neutrophil engraftment was 13 (range, 10–27) days in the aGVHD group and 15 (range, 11–23) days in the non-aGVHD group. The median time to platelet engraftment was 15 (range, 10–32) days in the aGVHD group and 15 (range, 10–45) days in the non-aGVHD group. No significant differences were observed in hematopoietic reconstitution between the two groups (P >0.05). Univariate analysis indicated that female patients were more susceptible to aGVHD (P = 0.007) (**Table 2**).

**Table 2:** Characteristics of patients with aGVHD vs. non aGVHD in training set.

Characteristics	aGVHD	Non aGVHD	*P* value
Gender, n			0.007
Male	10	39	
Female	19	21	
Age, median (range), y	27 (4–58)	34.5 (2.7–61)	0.207
Diseases, n			0.127
AML	10	30	
ALL	9	13	
MDS	4	10	
CML	2	6	
AA	4	1	
Donor type, n			0.095
HID	22	41	
MSD	3	15	
MUD	2	4	
UCBD	2	0	
Neutrophil engraftment, median (range), d	13 (10–27)	15 (11–23)	0.114
Platelet engraftment, median (range), d	15 (10–32)	15 (10–45)	0.487
CD34^+^cells, median (range), ×10^6^/kg	3.7 (0.1–5.3)	3.6 (0.9–8.2)	0.837
MNC, median (range), ×10^8^/kg	8.3 (0.2–14.1)	8.2 (1.6–18.3)	0.713
Engraftment syndrome, n			0.212
Yes	5	5	
No	24	55	
Sepsis, n			0.511
Yes	5	14	
No	24	46	

AML, acute myeloid leukaemia; ALL, acute lymphoid leukaemia; MDS, myelodysplastic syndrome; CML, chronic myeloid leukaemia; AA, aplastic anemia; HID,haploidentical donors; MSD, matched sibling donors; MUD, matched unrelated donors; UCBD, unrelated cord blood donors; MNC, mononuclear cells.

**Figure 1:**
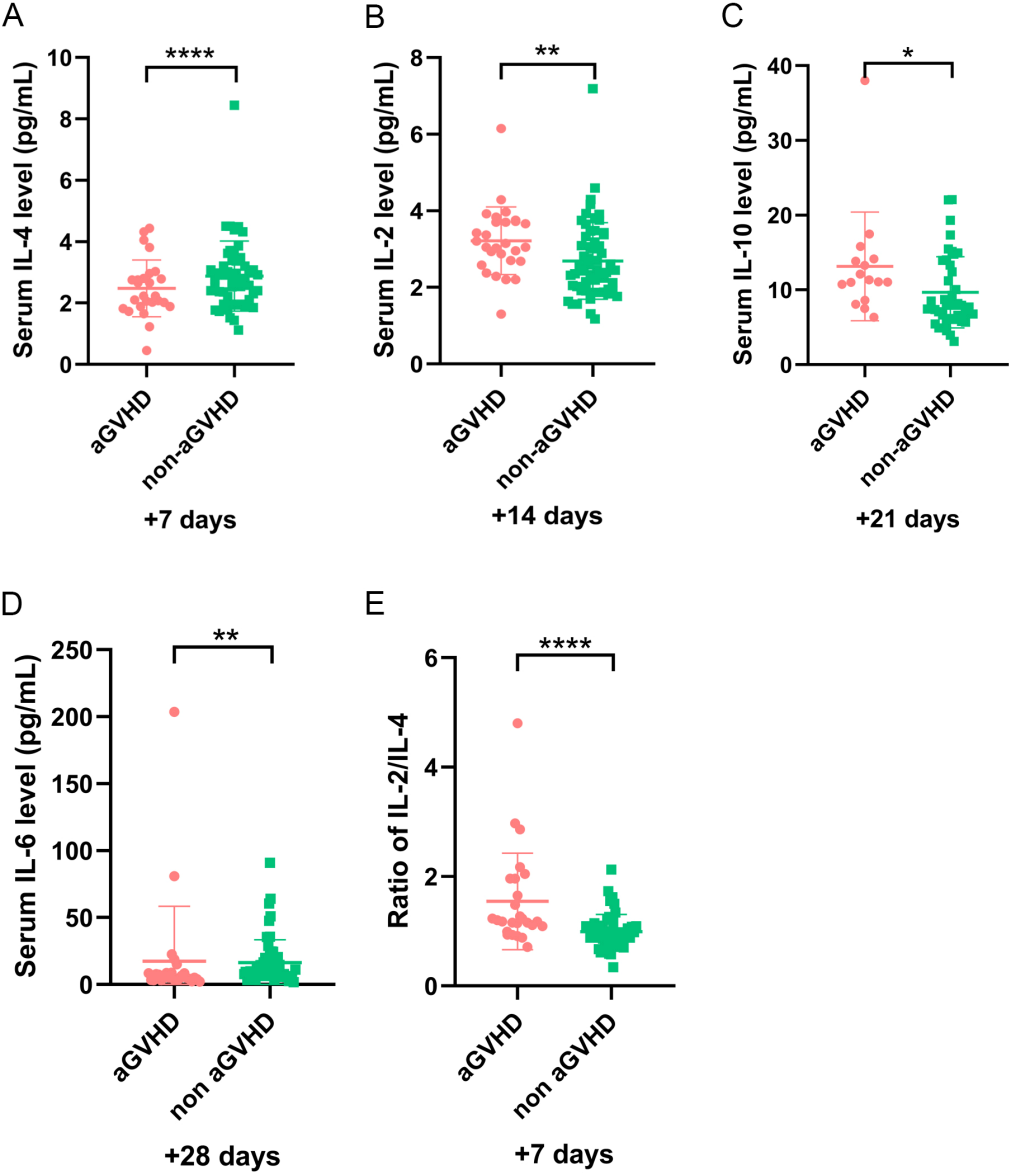
The level of cytokines at different time points in patients with and without aGVHD in training set. **(A)** The level of IL-4 on day +7, **(B)** IL-2 on day +14, **(C)** IL-10 on day +21, **(D)** IL-6 on day +28, and **(E)** IL-2/IL-4 ratio on day +7 in aGVHD and non aGVHD groups. *P<0.05, **P<0.01, ****P<0.0001.

In our study, five cytokines were detected, including IL-2, IL-4, IL-6, IL-10, and TNF-α. To further elucidate the role of cytokines, we explored their relationship with aGVHD-related events. Univariate analysis revealed that IL-4 on day +7 and IL-6 on day +28 significantly decreased in patients with aGVHD compared with those in the control group (P <0.001 and P = 0.003, respectively) (**Figures 1A, D**). The expression of IL-2 on day +14 and IL-10 on day +21significantly elevated in the aGVHD cohort (P = 0.004 and P = 0.023, respectively) (**Figures 1B, C**). Cytokine levels at other time points demonstrated no significant differences in patients with and without aGVHD (**Supplementary Table 1**).

**Figure 2:**
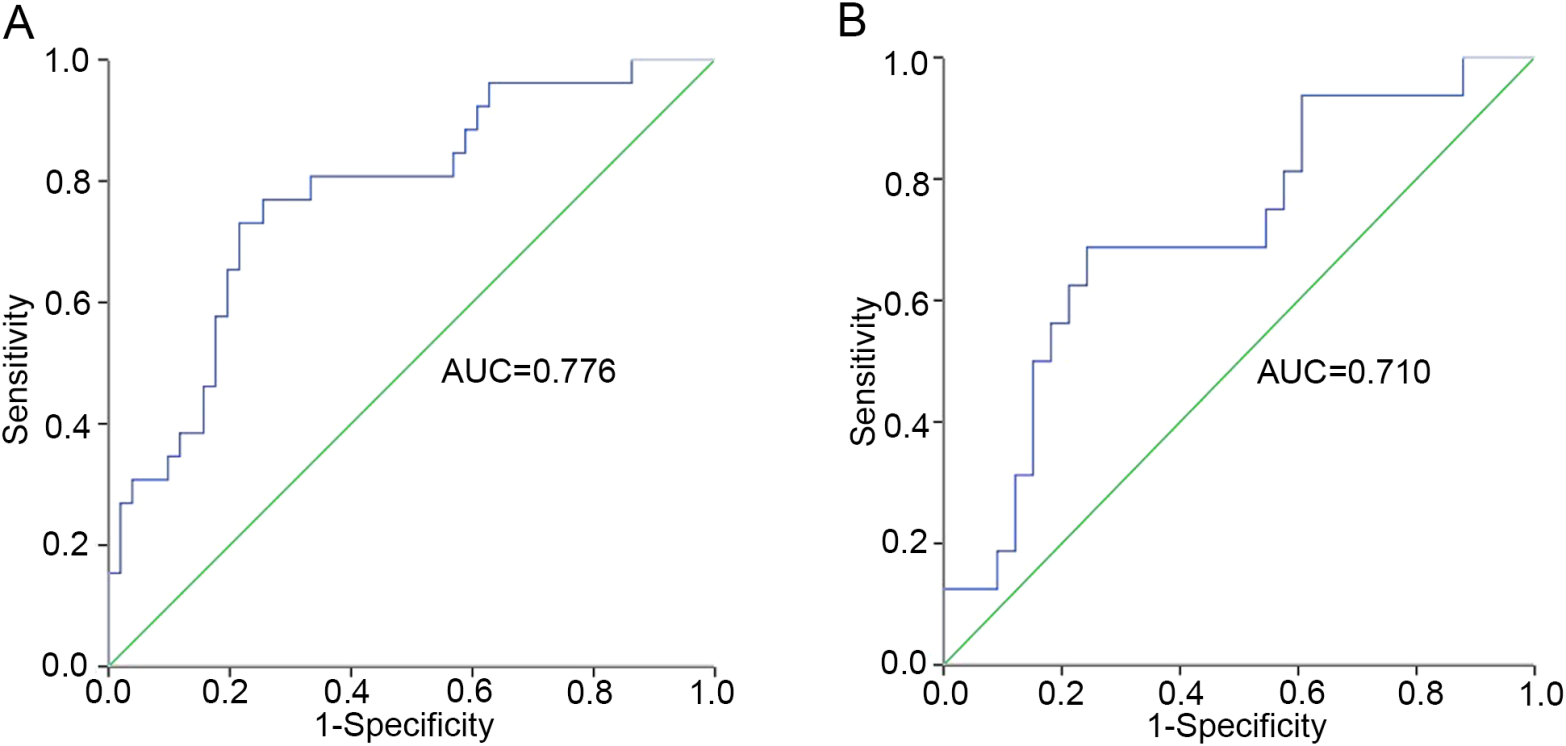
The cut-off value of IL-2/IL-4 ratio for patients in training set. **(A)** The whole cohort (n=89). **(B)** Patients excluding sepsis and ES cases (n=60).

To minimize variability in cytokine level detection among different laboratories, we investigated the relationship between IL-2/IL-4 ratio and aGVHD further. Our results demonstrated that elevated IL-2/IL-4 ratio may be associated with aGVHD onset. The ROC analysis identified an optimal cut-off of IL-2/IL-4 ratio on day +7 was 1.103 (sensitivity, 0.731; specificity, 0.784) (**Figure 2A**). Patients with IL-2/IL-4 ≥1.103 on day +7 had a significantly increased risk of aGVHD (P < 0.001; **Figure 1E**). Logistic regression analysis reveled that IL-2/IL-4 ratio ≥1.103 was associated with an 8.87-fold increased risk of aGVHD (OR: 9.870; 95%CI: 1.830–25.823; P <0.001).

#### 3.2.2 Influence of engraftment syndrome and sepsis on cytokines level

**Figure 3:**
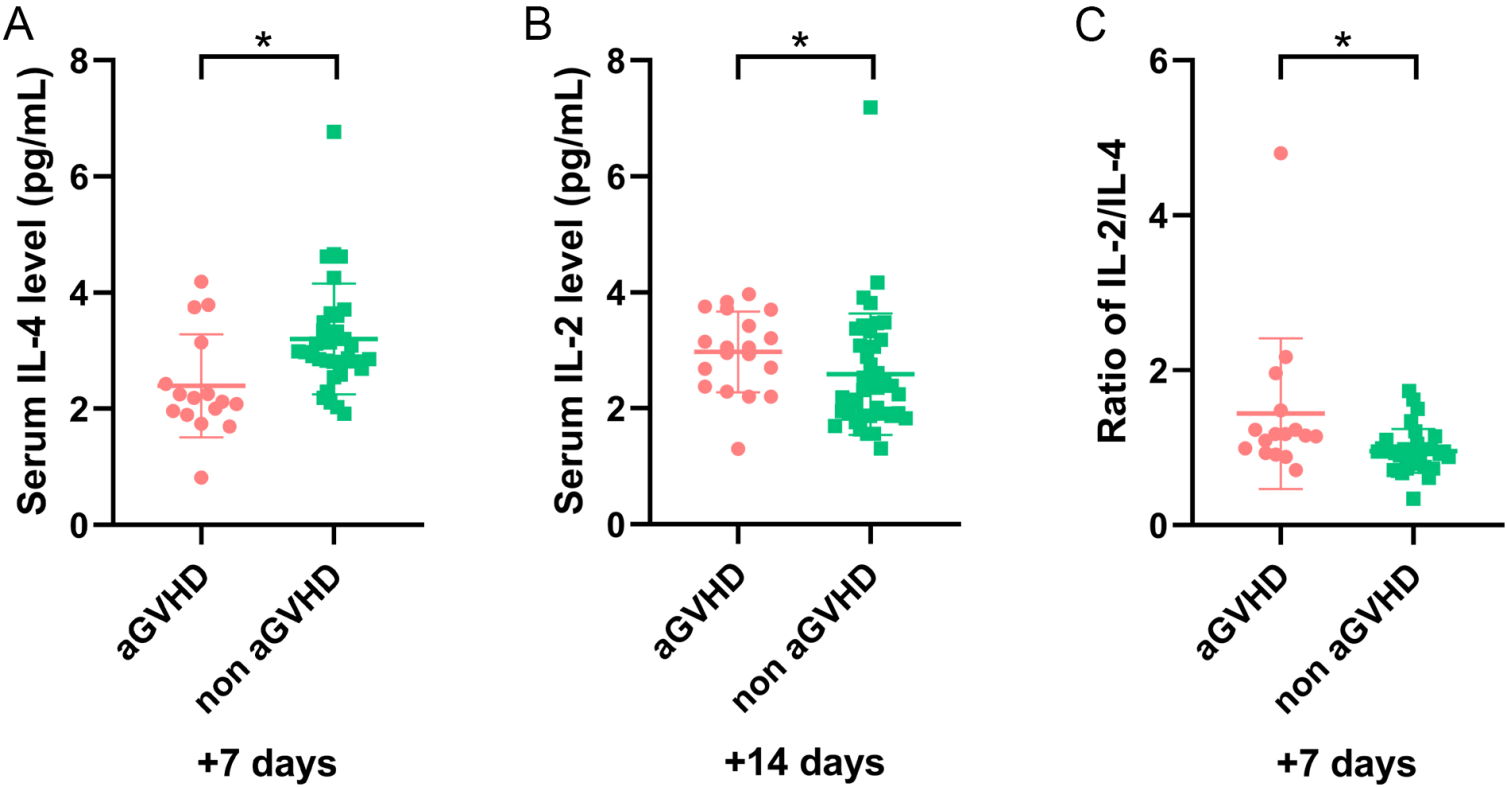
The level of cytokines in remaining patients (sepsis and ES cases were excluded) with and without aGVHD in training set. **(A)** The level of IL-4 on day +7, **(B)** IL-2 on day +14, and **(C)** IL-2/IL-4 ratio on day +7 in aGVHD and non aGVHD groups. *P<0.05.

Considering the effect of ES and sepsis on cytokines, we excluded 19 patients with sepsis and 10 patients with ES, and further reevaluated the remaining 60 patients in training set. Amongst these patients, 19 had aGVHD and 41 did not. Univariate analysis revealed that the IL-4 level on day +7 was significantly decreased, whereas IL-2 on day +14 increased in patients with aGVHD compared with that in the control group (P = 0.021 and P = 0.044, respectively) (**Figures 3A, B**). In particular, patients with higher IL-2/IL-4 ratios on day +7 were also more inclined to develop aGVHD (P = 0.018) (**Figure 3C**), and the cut-off was 0.989 (sensitivity, 0.688; specificity, 0.758) (**Figure 2B**). The IL-2/IL-4 ratio ≥0.989 suggested a 5.875-fold increased risk of aGVHD (OR: 6.875; 95%CI: 1.830–25.823; P = 0.004). No significant correlations were observed between cytokines levels and aGVHD onset.

**Figure 4:**
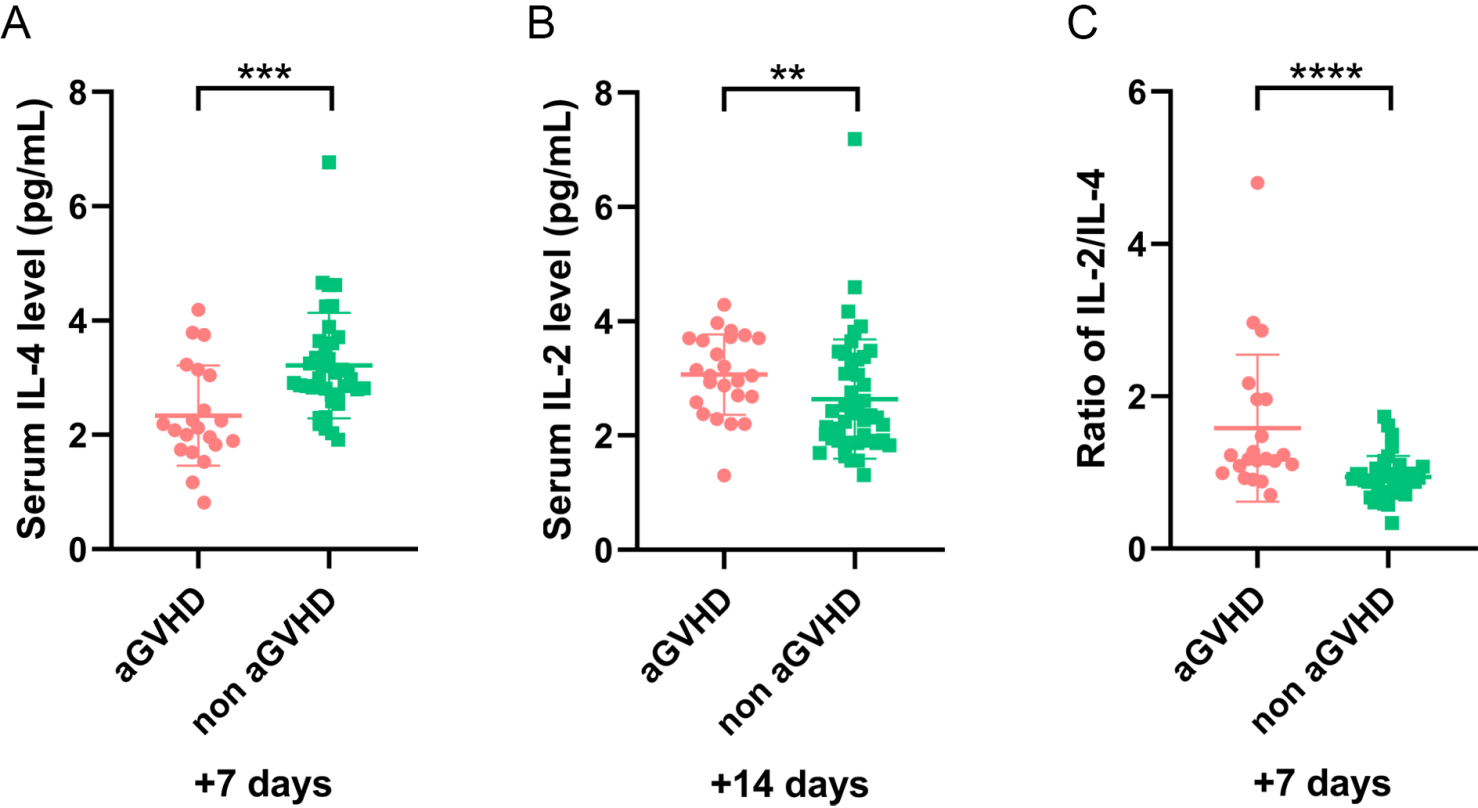
The level of cytokines in patients (sepsis cases were excluded) with and without aGVHD in training set. **(A)** The level of IL-4 level on day +7, **(B)** IL-2 on day +14, and **(C)** IL-2/IL-4 ratio on day +7 in aGVHD and non aGVHD groups. **P<0.01, ***P<0.001, ****P<0.0001.

To exclude the impact of sepsis on cytokines, 19 patients with sepsis were excluded, and the remaining 70 patients were analyzed further. Univariate analysis revealed that the IL-4 level on day +7 significantly decreased and IL-2 on day +14 increased in patients with aGVHD compared with those in the control group (P = 0.001 and P = 0.008, respectively) (**Figures 4A, B**). The IL-2/IL-4 ratio on day +7 was higher in the aGVHD group (P <0.001) (**Figure 4C**). After excluding 10 patients with ES alone, cytokines trends in the remaining 79 patients were consistent. Our findings suggested sepsis and ES may not impact these results.

In the subgroup analysis of 29 patients with aGVHD, excluding 5 with ES, the remaining 24 were evaluated, including 5 with sepsis. The median onset of sepsis was 10.8 (range, -1– +40) days. IL-2 levels on days +14 and +21 were significantly elevated in patients with sepsis than those in the control group (P = 0.021 and P = 0.020). Similarity, after excluding 5 patients with sepsis from the 29 patients with aGVHD, the remaining patients were evaluated, including 5 with ES. The median onset of ES was 11 (range, 8–13) days. Univariate analysis revealed no significant differences in cytokine levels between patients with or without ES.

In the 60 patients without aGVHD, after excluding 5 with ES, 55 patients were evaluated. Among these, 14 had sepsis, with a median onset of 0 (range, -2– +5) days. Our results showed that the expression of IL-10 on day +7 (P = 0.005), TNF-α and IL-6 on day +14 (P = 0.041 and P =0.010), and IL-6 on day +28 (P = 0.010) were higher in patients with sepsis than the control group. After excluding 14 patients with sepsis, further analysis showed no significant differences in cytokine levels between patients with or without ES (all P >0.05).

#### 3.2.3 Correlation between the IL-2/IL-4 ratio and aGVHD onset in the validation set

**Figure 5:**
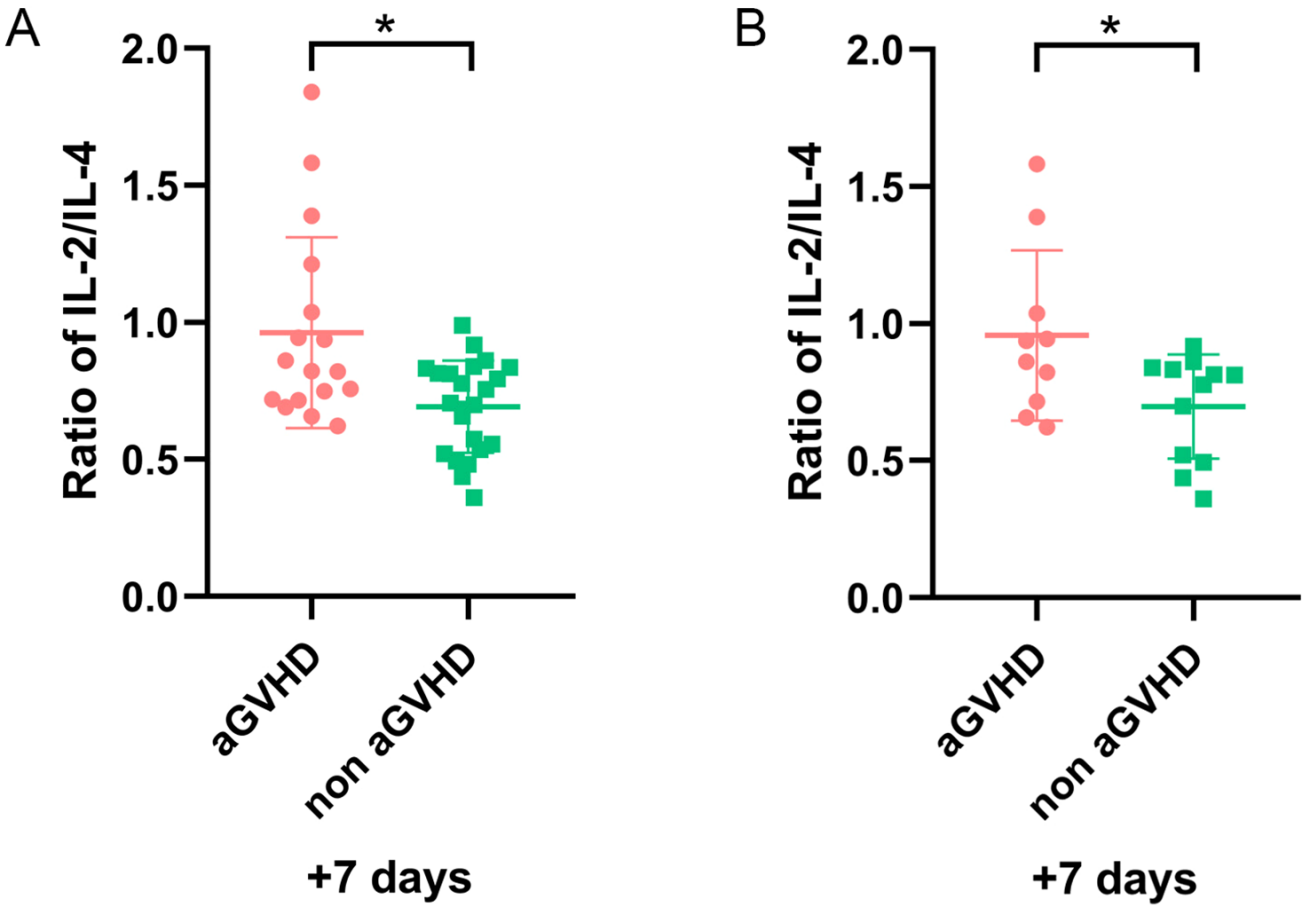
The ratio of IL-2/IL-4 on day +7 for patients with and without aGVHD in validation set. **(A)** The whole cohort (n=40). **(B)** Patients excluding sepsis and ES cases (n=21). *P<0.05.

In the validation set, 17 patients developed aGVHD. The median age was 28.8 (range, 9–57) years, and 41.2% were male. Another 23 patients without GVHD included 14 males and 9 females, with a median age of 28.6 (range, 10–57) years. Univariate analysis showed no significant differences between the two groups (**Table 3**). Importantly, patients with a higher IL-2/IL-4 ratio on day+7 were also susceptible to aGVHD (P = 0.011) (**Figure 5A**).

**Table 3:** Characteristics of patients with aGVHD vs. non aGVHD in validation set.

Characteristics	aGVHD	Non aGVHD	*P* value
Gender, n			0.302
Male	7	14	
Female	10	9	
Age, median (range), y	28.8 (9–57)	28.6 (10–57)	0.965
Diseases, n			0.588
AML	8	10	
ALL	6	6	
MDS	1	2	
CML	0	1	
AA	2	4	
Donor type, n			0.829
HID	14	20	
MSD	2	2	
MUD	0	1	
UCBD	1	0	
Neutrophil engraftment, median (range), d	14 (12–21)	15 (11–21)	0.705
Platelet engraftment, median (range), d	14 (13–40)	17 (12–30)	0.211
CD34^+^cells, median (range), ×10^6^/kg	4.4 (0.2–9.0)	4.3 (1.7–8.4)	0.533
MNC, median (range), ×10^8^/kg	6.0 (0.3–10.8)	6.2 (4.8–11.4)	0.416
Engraftment syndrome, n			0.432
Yes	7	6	
No	10	17	
Sepsis, n			0.401
Yes	1	5	
No	16	18	

AML, acute myeloid leukaemia; ALL, acute lymphoid leukaemia; MDS, myelodysplastic syndrome; CML, chronic myeloid leukaemia; AA, aplastic anemia; HID,haploidentical donors; MSD, matched sibling donors; MUD, matched unrelated donors; UCBD, unrelated cord blood donors; MNC, mononuclear cells.

After excluding 6 patients with sepsis and 13 patients with ES, the remaining 21 patients were analyzed. Among them, 9 had aGVHD, and 12 did not. Our results demonstrated a higher IL-2/IL-4 ratio remained a strong predictor of aGVHD onset (P = 0.043) (**Figure 5B**).

#### 3.2.4 Correlation between serum cytokines and the severity of aGVHD

**Figure 6:**
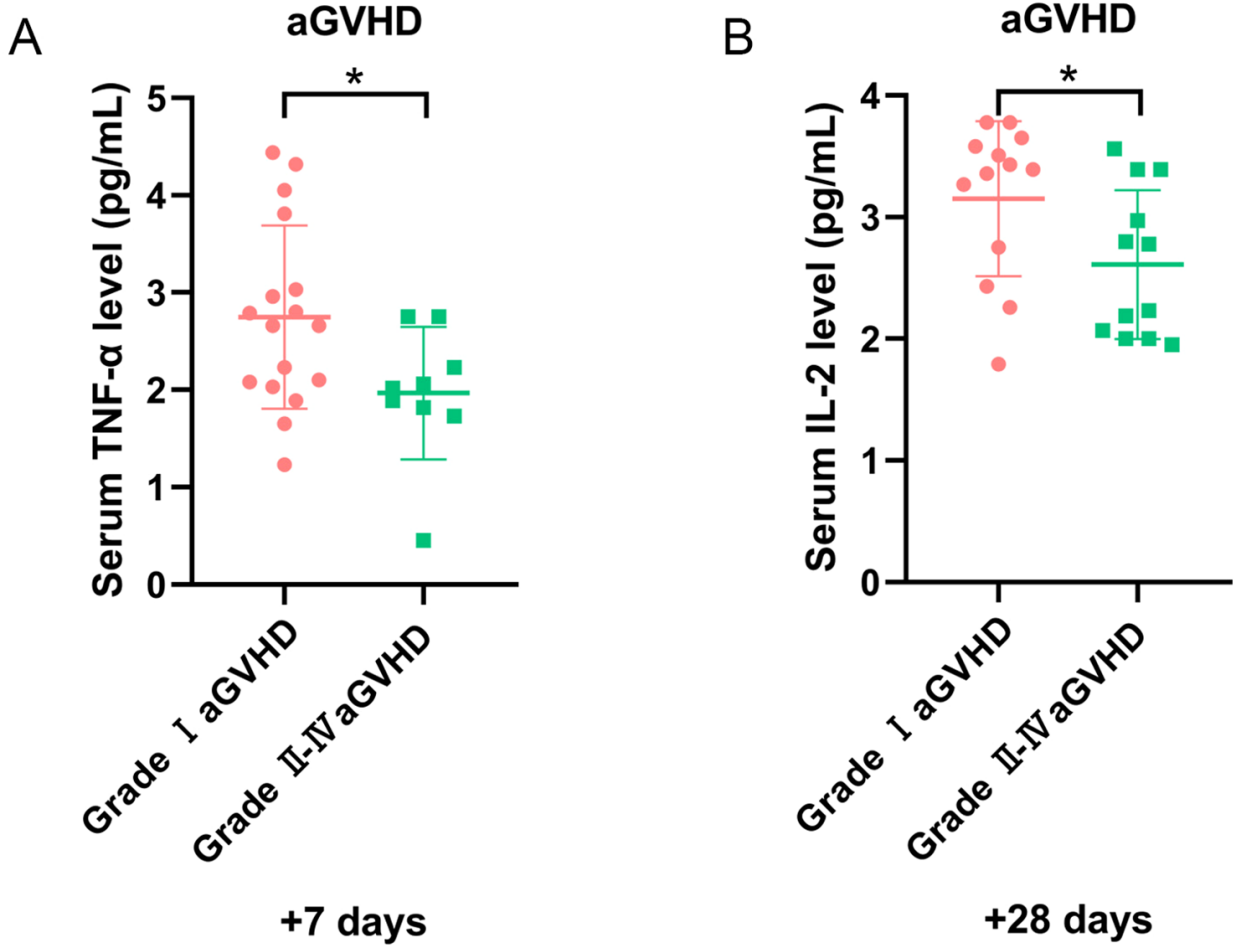
The level of cytokines for patients with aGVHD in different grades. **(A)** The level of TNF-α on day +7, and **(B)** IL-2 on day +28 in grade I and II-IV aGVHD groups. *P<0.05.

All grades aGVHD developed in 29 patients, with grade II–IV aGVHD in 12 cases. Organ involvement included skin (20 cases), gastrointestinal (5 cases), liver (1 case), concurrent skin/gastrointestinal (3 cases). Univariate analysis demonstrated increased TNF-α on day +7 and IL-2 on day +28 in the grade I aGVHD group (P = 0.038 and P = 0.039, respectively) (**Figures 6A, B**). After excluding 10 patients with sepsis or ES, 12 had grade I aGVHD and 7 had grade II–IV aGVHD. Among them, 13 had skin aGVHD, 1 had liver aGVHD, 3 had gastrointestinal aGVHD, and 2 had concurrent skin/gastrointestinal aGVHD. No significant differences in cytokine levels were observed between patients with grade I and grade II–IV aGVHD.

#### 3.2.5 Correlation between serum cytokines and therapeutic efficacy of aGVHD

Twenty-two of 29 patients received corticosteroids as first-line treatment, whereas others received topical or low-dose corticosteroids. Fifteen of 22 patients were sensitive to steroids and achieved complete remission (CR), with a CR rate of 68.2%. CR rates were 90.9% (10/11) in grade I aGVHD and 45.5% (5/11) in grade II–IV aGVHD. After excluding patients with sepsis or ES, 14 of 19 patients with aGVHD received steroids as first-line treatment. The CR rate in this subgroup was 76.9% (11/14), and 66.7% (2/3) of patients with no remission (NR) had grade II–IV aGVHD. Patients with a poor response to standard first-line treatment were given basiliximab, tacrolimus, ruxolitinib, MMF, or mesenchymal stem cells. Our results revealed cytokine levels were comparable between patients achieved CR and those did not.

## 4 Discussion

Allo-HSCT is a potential cure for hematopoietic malignancies. Despite improved effectiveness of allo-HSCT, aGVHD remains a major complication, making early diagnosis and treatment crucial to prognosis. Certain cytokines levels are associated with aGVHD onset, severity, and steroid sensitivity (8, 20, 24). ES and sepsis are common post-HSCT complications; ES is a non-infectious complication characterized by the presence of non-infectious fever, diarrhea, skin rash, pulmonary infiltration, and edema. ES incidence in patients who underwent allo-HSCT ranges from 10 to 77% (25, 26). Pro-inflammatory cytokines and immune response dysregulation drive ES pathogenesis. In addition, neutropenia and immunosuppressive therapy increase infectious complications, including sepsis. During sepsis, inflammation and immunosuppression may occur sequentially or concurrently (27). Both sepsis and ES substantially affect cytokine levels, complicating aGVHD diagnosis and treatment.

Owing to experimental limitations, only five cytokines were tested: IL-2, IL-4, IL-6, IL-10, and TNF-α. IL-2, IL-6, and TNF-α are pro-inflammatory cytokines, whereas IL-4 and IL-10 are anti-inflammatory cytokines (28, 29). In the pathogenesis of aGVHD, IL-2 promotes the activation and proliferation of alloreactive T cells and T helper type 1 (Th1), and inhibits regulatory T cells (Tregs) function, thereby driving tissue damage and further inflammation (28, 29). IL-2 level in patients with aGVHD was significantly elevated and gradually decreased with treatment. Additionally, higher sIL-2R levels was higher in patients with III/IV aGVHD than I/II aGVHD suggests its relation to the severity and potential as an early monitoring indicator for aGVHD (30, 31). IL-6 level was also highly elevated in patients with III/IV aGVHD, though commonly expressed in severe inflammation (24, 28, 29, 32). Conversely, IL-4 and IL-10 protect against aGVHD by polarizing immune responses toward T helper type 2 (Th2) differentiation, suppressing Th1/Th17 pathways, and potentiating Treg-mediated tolerance (28, 29).

Our study revealed that lower IL-4 on day +7 and higher IL-2 on day +14 in aGVHD group, consistent with previous studies. IL-10 increased on day +21 and IL-6 decreased on day +28 in patients with aGVHD, possibly related to immune recovery and anti-inflammatory activation. For the first time, we observed that patients with a higher IL-2/IL-4 ratio on day +7 correlated with aGVHD susceptibility. This finding was further validated in an independent cohort. In our study, the temporal disparity in patients enrollment resulted in differential distributions of CD34^+^ cells and MNC between the training and validation set. However, in both groups, CD34^+^ cells and MNC were comparable in patients with and without aGVHD, and a higher IL-2/IL-4 ratio was identified as the early predictor for aGVHD onset, underscoring the applicability and stability of this ratio further. The selection of this ratio was driven by the slightly elevated level of IL-2 on day +7, without statistical difference, while IL-4 decreased significantly in aGVHD group. To predict aGVHD onset earlier, we chosen “day +7” as the optimal timepoint. The IL-2/IL-4 ratio may reflect the balance between pro- and anti-inflammatory status, thereby as a potential predictor for aGVHD is feasible. Furthermore, this ratio may help minimize variability and standardize cytokine detection among different laboratories, improving accuracy. While the sensitivity and specificity of this ratio were suboptimal, its predictive value could potentially be enhanced through integrating with immune reconstitution parameters and established biomarkers (e.g., ST2, TNFR1, or Reg3α). Clinically, we did examine immune cell subsets and biomarkers at different time points; however, the available data were incomplete for comprehensive analysis. Further prospective studies with larger cohorts are needed to confirm these results and optimize the predictive accuracy.

ES and aGVHD are common complications following HSCT, and ES is associated with neutrophil recovery. These complications arise from innate immune hyperactivity and a pro-inflammatory cytokine storm (25). Similar clinical symptoms and underlying pathophysiology make distinguishing between ES and aGVHD challenging (26). A meta-analysis demonstrated a 35.4% cumulative incidence of ES after allo-HSCT, with higher odds of aGVHD and non-relapse mortality (NRM) in these patients (33). Another meta-analysis demonstrated that ES tripled the odds of developing aGVHD in the ES group when compared to the control group (34), aligning with previous studies (35–38); however, our results did not confirm this. Patients with ES had higher serum levels of IL-1β, IL-6, IL-12, TNF-α, and IFN-γ than those with aGVHD (26, 39). In our training set, 29 patients developed aGVHD, including 5 with ES. However, among these patients, no significant differences in cytokine levels were observed, suggesting ES may not affect cytokines in patients with aGVHD. These findings were inconsistent with previous studies (26, 39), suggesting the need for larger patient samples to confirm findings.

Due to immunosuppression, patients who underwent allo-HSCT frequently develop infections, and in severe cases, sepsis. Sepsis is a severe clinical syndrome related to the immune response to infection. The innate and adaptive immune systems release inflammatory cytokines early in sepsis to eliminate foreign pathogens, such as IL-6, TNF-α, and IL-1β. Among these cytokines, IL-6 is a key biomarker and prognostic indicator of sepsis. Moreover, sepsis-related anti-inflammatory cytokines primarily include IL-4, IL-10, and IL-37. Under physiological conditions, the dynamic balance between pro-inflammatory and anti-inflammatory responses maintains immune homeostasis. However, during sepsis, this balance is disrupted. The upregulated expression of pro-inflammatory cytokines released by inflammatory cells and activation of the complement and coagulation systems result in excessive inflammation, leading to cytokine storms and high mortality (27). To explore the effect of sepsis on cytokines in patients with aGVHD, a subgroup analysis was conducted. In our study, excluding 5 patients with ES, 24 patients with aGVHD were analyzed. Among them, five had sepsis, with an incidence of 20.83%. Our results suggested IL-4 levels and the IL-2/IL-4 ratio on day +7 were higher in patients with sepsis than those in the control group; however, the difference was not significant. Nevertheless, IL-2 levels were markedly higher in patients with sepsis on days +14 and +21, suggesting sepsis influences IL-2 expression, consistent with those in patients with aGVHD, without affecting result interpretation.

Both sepsis and ES may influence cytokine levels. To minimize potential confounding effects, we excluded patients with sepsis or ES and reevaluated the remaining individuals. The expression of IL-2, IL-4 and the IL-2/IL-4 ratio in these patients remained consistent, validating our previous observations. Notably, the IL-2/IL-4 ratio on day +7 in patients with aGVHD showed minimal change, regardless of sepsis presence. This suggested that the IL-2/IL-4 ratio was a more accurate predictor of aGVHD, aiding in early intervention.

The incidence of grade II–IV aGVHD after allo-HSCT is approximately 13%–47% (2, 40). Despite improved overall survival (OS) in patients with aGVHD over time (41, 42), mortality remains high for severe cases, with early mortality up to 46% (43). One-year OS is 70% in patients with grade II aGVHD and 40% in patients with grade III–IV aGVHD (2, 41). Most studies link cytokines to aGVHD severity; the absence of such findings in our studies may be attributed to the predominance of patients with grade I aGVHD and a limited sample size.

In our study, the response rate for patients with grade II–IV aGVHD was 45.5%, consistent with that reported in literature. However, the factors affecting efficacy remained controversial. In the REACH2 trial, various markers were measured, including immune cell subtypes, inflammatory cytokines, and proteins associated with aGVHD-damaged target organs. Baseline and day +14 models were first developed to identify variables affecting response probability among patients with SR-aGVHD in a randomized trial (44). OS was poor for these patients, highlighting the need for effective second-line treatment. Our results revealed cytokine levels were comparable between patients achieved CR and those did not. Future studies with expanded cohort are needed to confirm these results and establish clinical applications.

In conclusion, a higher IL-2/IL-4 ratio on day +7 was an early predictor of aGVHD post-HSCT, even with sepsis or ES. Nevertheless, whether these five cytokines could predict aGVHD severity or therapeutic efficacy remain unclear. Owing to the inherent retrospective analysis bias and a limited sample of eligible patients, results should be interpreted cautiously and prospective validation in larger cohorts is warranted to validate these preliminary results.

## Data Availability

The original contributions presented in the study are included in the article/**Supplementary Material**. Further inquiries can be directed to the corresponding author/s.
