# Supplementary material for: A higher ratio of IL-2/IL-4 may be an early predictor of acute graft-versus-host disease after allogeneic hematopoietic stem cell transplantation
Source: Front Immunol. 2025 Jul 11;16:1620761. doi: 10.3389/fimmu.2025.1620761 (PMC12289477; doi:10.3389/fimmu.2025.1620761)
Supplement: Supplementary file 1 [file Table1.docx]

**Supplemental Table 1: Cytokines level at different time points in training set**

| **Cytokines** | **Time points** | **aGVHD** | **Non aGVHD** | ***P* value** |
| --- | --- | --- | --- | --- |
| TNF-α | +7 | 2.23(1.89–2.84) | 2.71(2.11–3.37) | 0.129 |
|  | +14 | 2.09(1.78–2.79) | 2.33(1.74–2.83) | 0.379 |
|  | +21 | 2.21(1.48–2.80) | 2.175(1.72–3.31) | 0.497 |
|  | +28 | 2.51(1.68–2.83) | 2.44(1.79–3.26) | 0.576 |
| IL-10 | +7 | 10.74(8.59–13.87) | 11.52(8.97–16.25) | 0.413 |
|  | +14 | 8.50(5.53–13.36) | 7.72(6.14–10.5) | 0.579 |
|  | +21 | 13.72(7.86–27.76) | 14.35(7.25–36.01) | 0.023* |
|  | +28 | 5.39(4.02–8.62) | 7.48(6.07–10.44) | 0.930 |
| IL-6 | +7 | 37.32(18.89–56.89) | 32.88(20.31–58.63) | 0.923 |
|  | +14 | 16.58(12.31–28.06) | 16.33(11.18–45.96) | 0.784 |
|  | +21 | 13.72(7.86–27.76) | 14.35(7.25–36.01) | 0.767 |
|  | +28 | 5.39(4.02–8.62) | 10.71(6.81–17.20) | 0.003* |
| IL-2 | +7 | 3.40(2.46–3.85) | 2.87(2.30–3.52) | 0.314 |
|  | +14 | 3.10(2.69–3.72) | 2.43(1.95–3.33) | 0.004* |
|  | +21 | 3.26(2.27–3.55) | 2.49(1.80–3.51) | 0.235 |
|  | +28 | 2.97(2.21–3.47) | 2.57(1.99–3.44) | 0.344 |
| IL-4 | +7 | 2.16(1.82–3.07) | 2.98(2.59–3.64) | 0.000* |
|  | +14 | 2.52(1.89–3.15) | 2.33(1.87–3.28) | 0.942 |
|  | +21 | 2.26(1.98–3.32) | 2.43(1.92–3.25) | 0.979 |
|  | +28 | 2.36(1.81–3.04) | 2.51(1.98–3.55) | 0.280 |
| IL-2/IL-4 | +7 | 1.19(1.07–1.96) | 0.95(0.79–1.09) | 0.000* |
